# A Novel Dual Antibody Staining Assay to Measure Estrogen Receptor Transcriptional Activity

**DOI:** 10.1007/s10895-020-02635-7

**Published:** 2020-11-17

**Authors:** Freek van Hemert, Christa Dam-de Veen, Sil Konings, John van der Ven, Anja van de Stolpe

**Affiliations:** Precision Diagnostics, Philips Research, Eindhoven, The Netherlands

**Keywords:** Estrogen receptor, Transcription factor complex, ER pathway, Immunocytochemistry, Pathway signalling

## Abstract

Activity of the canonical estrogen receptor (ER) pathway is equivalent to functional activity of the nuclear ER transcription factor. Monoclonal antibodies (MoAbs) that identify nuclear ER in cells and tissue samples are frequently used to assess ER transcriptional activity, however, it remains unclear if this approach is sufficiently predictive of ER pathway activity. This study uses ER-positive breast cancer cell lines (MCF7 and T47D) in which ER transcriptional activity was quantified using an mRNA-based ER pathway activity assay. The relationship between ER activity and nuclear ER staining with ER MoAbs was then investigated. Confirming earlier findings, the results show that while the presence of ER in the cell nucleus is a prerequisite for ER activity, it is not predictive of ER transcriptional activity. There were remarkable differences in the behaviours of the antibodies used in the study. EP1 and 1D5 showed reduced nuclear staining when ER was transcriptionally active, while staining with H4624 was independent of ER activity. To improve discrimination between active and inactive nuclear ER based on ER staining, a method was developed which consists of dual ER MoAb immunofluorescent staining, followed by generation of a digital image with a standard digital pathology scanner. Then a cell nucleus detection algorithm and *per cell* calculation of the nuclear H4624/EP1 fluorescence intensity ratio was applied, where a high H4624/EP1 ratio predicts an active ER pathway. With this method, the EP1 and 1D5 antibodies are interchangeable. We hypothesize that the transcriptional activation of ER hides the epitope recognized by MoAbs EP1 and 1D5, while H4624 binds an ER epitope that remains accessible during ER pathway activation. The method described in this study should add substantial value to the assessment of ER pathway activity for biomedical research and diagnostics.

**Electronic supplementary material:**

The online version of this article (10.1007/s10895-020-02635-7) contains supplementary material, which is available to authorized users.

## Introduction

The estrogen receptor (ER) signal transduction pathway plays an important role in physiological processes such as the menstrual cycle, as well as in the pathophysiology of a variety of diseases such as breast cancer. Simplified, the signalling pathway consists of an estrogen ligand that binds the estrogen receptor, resulting in receptor dimerization and recruitment of co-activator molecules. This initiates the transcription of ER target genes that contain an estrogen response element in their promoter region. These target gene mRNAs are translated into proteins which change the behaviour of the cell, for example by driving cell division or differentiation. Other signalling pathways may crosstalk with the ER pathway, increasing the complexity of this cellular mechanism. The ability to measure the activity of the ER signalling pathway is important to unravel these complex signalling and intracellular crosstalk mechanisms. Moreover, measurement of this pathway is important in the breast cancer field for drug development and as a diagnostic method to make better decisions on the use of hormone therapy.

ER antibody staining is frequently used for research and diagnostics purposes, wherein positive nuclear ER staining is considered indicative of an active ER pathway. However, it is unclear how well nuclear ER staining is able to measure ER transcriptional activity. For example, up to half of breast cancer patients selected for hormonal therapy based on positive ER staining do not respond, suggesting that ER expression does not necessarily imply an active ER pathway in cancer tissue [1, 2].

To address this issue, we have previously developed an assay which allows quantitative measurement of ER pathway activity based on measuring ER target gene mRNA levels followed by a knowledge-based Bayesian computational model [1]. Evaluation of this ER pathway assay on a large number of ER positive and negative breast cancer samples as well as three independent clinical patient cohorts treated with neoadjuvant hormonal therapy provided evidence that nuclear ER staining is required, but not sufficient, for ER pathway activity [1, 3].

Since it remains important to be able to assess ER pathway activity on cell and tissue samples without the need to disrupt the cells to extract RNA, we here describe a fluorescent dual antibody ER staining assay, which improves prediction of the ER transcriptional activity state in cells on a cytology or tissue slide. The staining assay provides complementary information to the mRNA-based ER pathway activity assay, specifically spatial information on cellular ER pathway activity within an intact tissue architecture.

## Methods

### Cell Culture

Human epithelial cell breast cancer lines, MCF7 (HTB-22, ER+), T47D (HTB-133, ER+), and MDA-MB-231 (HTB-26, ER-), were purchased from ATCC (Washington, USA). Cells were cultured at 37 °C, 5% CO_2_, 95% humidity, in appropriate culture medium (Gibco, Carlsbad, California, USA). MCF7 and MDA-MB-231 were cultured in Dulbecco’s Modified Eagles Medium (DMEM) and T47D was cultured in Roswell Park Memorial Institute medium (RPMI). Medium was supplemented with 1% penicillin/streptomycin, 10% fetal bovine serum (FBS) and 1% Glutamax (Gibco, Carlsbad, California, USA). To generate ER pathway-inactive and ER pathway-active breast cancer cells, the cells were first deprived from estrogen for two days and subsequently stimulated with estradiol, according to Katzenellenbogen et al. and as described before [1, 4]. In brief, prior to estradiol stimulation, MCF7 cells were cultured for 48 h in phenol red-free culture medium, supplemented with 10% heat-inactivated and charcoal-treated fetal bovine serum (FBS) and 1% Glutamax. Subsequently, cells were stimulated with estradiol (E2, 10 nM, Sigma-Aldrich, Saint Louis, USA) or vehicle (DMSO, Sigma-Aldrich, Saint Louis, USA) for 16 h. These experiments were performed by BioDetection Systems (Amsterdam, The Netherlands).

For fluorescent ER protein staining experiments the same estrogen deprivation and stimulation protocol was followed, except that the cells were cultured on fibronectin-coated coverslips for various timepoints as indicated. For the dual fluorescent staining assays, fixation and staining were performed at 0 and 30 min E2 incubation.

### Immunofluorescent ER Staining

Cells were fixated with 4% pH neutral buffered formaldehyde (Boom BV, Meppel, The Netherlands) for 15 min, permeabilized and blocked with 0.2% Triton X-100/1% bovine serum albumin (BSA)/PBS solution for 20 min at room temperature (RT). Coverslips were rinsed, incubated with primary antibody for one hour in a humidified environment at RT, rinsed, incubated with secondary antibody for 30 min in a humidified and dark environment at RT, rinsed, and finally mounted on microscope slides with Vectashield Hard Set mounting medium containing DAPI (H-1500, Vector laboratories, Burlingame, California, USA).

Primary antibodies used were monoclonal mouse anti-human estrogen receptor-α 1 (1D5), monoclonal mouse anti-human estrogen receptor-α 2 (EP1), monoclonal rabbit anti-human estrogen receptor-α (H4624) and polyclonal rabbit anti-human estrogen receptor- α (H-184). EP1 and 1D5 MoAbs are routinely used in clinical pathology. Secondary antibodies used were goat anti-mouse ATTO-555 (1/200 dilution, Invitrogen, Carlsbad, California, USA) and/or goat anti-rabbit ATTO-633 (1/200 dilution, Invitrogen Carlsbad, California, USA) (for details, see Supplemental Table 1). For dual antibody ER staining, primary monoclonal antibodies (MoAb) from mouse and rabbit were used in combination. The ER negative MDA-MB-231 cell line was used as negative control for staining with ER MoAbs.

### Measurement of Functional ER Pathway Activity

Following deprivation and stimulation with estradiol, RNA was isolated using the Nucleospin RNA isolation kit (Macherey-Nagel)(BioDetection Systems, Amsterdam). Affymetrix HGU133Plus2.0 microarray analysis was performed by Service XS (Leiden, The Netherlands). ER pathway activity scores were calculated from the Affymetrix microarray expression data using the ER pathway activity assay as described before [1, 3]. In brief, the ER pathway assay infers transcriptional activity of ER from mRNA levels of a number of high-evidence ER target genes, in this case measured by Affymetrix microarrays. The calculated ER transcriptional activity score is inferred as equivalent of the ER pathway activity score. Activity scores are presented on a log2 odds scale [5, 6].

### Cell Image Analysis and Nuclear ER Staining Quantification Algorithm

Stained coverslips were scanned on a 3D Histech Pannoramic MIDI system equipped with a 20x objective and a CIS 3CCD camera. For dual antibody ER staining, fluorescence detection was performed in separate channels. For the DAPI channel 25 millisecond (ms) exposure without digital gain was used, for the TRITC channel 150 ms exposure without digital gain, and for the Cy5 channel 175 ms exposure with digital gain setting 3. Data were stored in MIRAX file format and converted to RGB TIFF files. Algorithms for analysis of digital images and quantification of immunofluorescent staining intensities were developed using MATLAB 2012b (The MathWorks Inc., Natick, MA, 2000, United States). Digital image analysis consisted of: (1) image pre-processing; (2) cell nucleus detection; (3) cell membrane location estimation; and (4) quantification of immunofluorescence staining intensity in detected nuclei. Details are described in Supplementary Methods.

### Fluorescent Staining Data Analysis

For all cells on a sample slide, nuclear immunofluorescence intensity values were calculated and data were analysed and plotted using the Python3 (Python Software Foundation, https://www.python.org/) modules Pandas [7], matplotlib [8], Seaborn [9] and scikit-learn [10]. Immunofluorescence intensity results were quantitatively compared using intensity histograms.

### Prediction of ER Activity from Dual ER MoAb Immunofluorescent Staining Data

The ER activity status per individual cell was calculated based on a comparison between nuclear fluorescent staining intensity of EP1 and H4624 MoAb. H4624 staining intensity represented the total amount of nuclear ER, that is, both functionally active and inactive, while EP1 (or alternatively 1D5) staining intensity represented only the amount of inactive ER. To predict ER activity, H4624 and EP1 fluorescent intensity signals were used to generate a H4624/EP1 staining intensity ratio for each individual cell nucleus. Since the polyclonal H184 ER antibody also stains ER independent of its activity state, a similar ratio to predict ER activity may be calculated by replacing H4624 staining intensity with H184 staining intensity.

This ratio number was used to generate a Receiver Operating Curve (ROC) curve for H4624/EP1 ratio-based prediction of nuclear ER activity. In addition, ROC curves were generated based on the staining intensities of individual antibodies.

## Results

### Nuclear ER Staining in ER Positive Breast Cancer Cells with an Inactive or Active ER

**Fig. 1:**
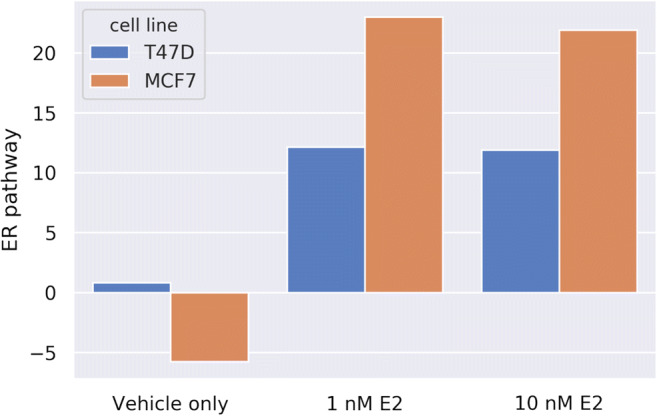
Breast cancer cell lines (T47D, MCF7) were deprived of estradiol for 48 h and subsequently stimulated with 1 or 10 nM estradiol, respectively, for 16 h. RNA was isolated and ER pathway activity was measured using Affymetrix U133P2.0 microarray expression analysis (see Methods for the used method). The ER pathway activity score is indicated as the log2 of the odds ratio of the probability that the ER pathway is active

We first investigated the relationship between nuclear ER staining and transcriptional ER activity. The ER-positive MCF7 and T47D breast cancer cell lines were used as in vitro model systems to measure ER pathway activity. Estrogen deprivation in these cells resulted in a low log2 odds ER pathway activity score (associated with an inactive ER pathway), while estradiol stimulation resulted in an increase in the ER pathway activity score (Fig. 1).

**Fig. 2:**
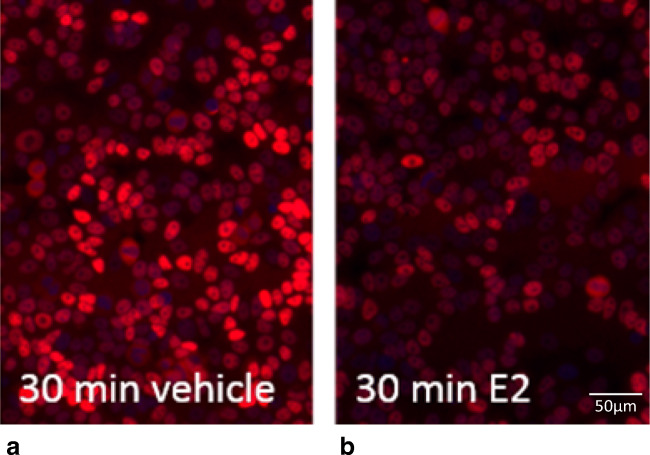
Nuclear ER staining using MoAB EP1 in breast cancer cells with an inactive or active ER pathway. A. Estrogen depleted MCF7 cells stimulated with vehicle (DMSO) for 30 min. B. Estrogen depleted MCF7 cells stimulated with 10 nM E2 (estradiol) for 30 min. The images are representative selections from the images that were quantified and used to create fig. 4. Magnification 200x (20x objective)

Immunofluorescent ER staining was subsequently performed using the EP1 MoAb on MCF7 cells in which the ER pathway was either inactive or stimulated with estradiol. EP1 showed specific nuclear ER staining in estrogen-deprived cells with an inactive ER pathway, while nuclear ER staining intensity decreased after activation of the ER pathway with estradiol (Fig. 2). This semi-quantitative analysis suggests that nuclear ER expression was present despite the absence of transcriptional activity, and that the induction of ER transcriptional activity unexpectedly reduced nuclear ER staining specifically with EP1. Based on this observation we proceeded to systematically quantify the relationship between ER transcriptional activity and immunofluorescent ER staining.

### Development of an Algorithm for Quantitative Assessment of Nuclear ER Staining per Cell

**Fig. 3:**
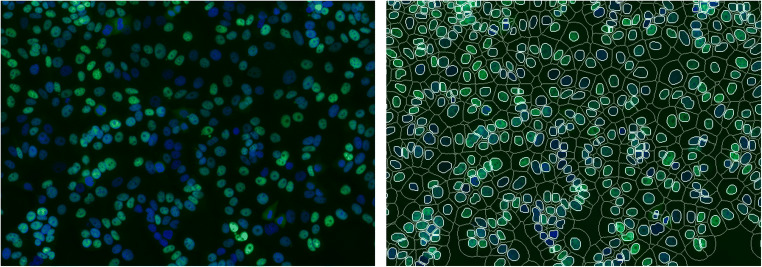
The nucleus detection algorithm reliably detects cell nuclei. **a**: Cells were cultured on a glass coverslip, routinely fixated and nuclei were stained with DAPI. **b**: The slide with the coverslip was scanned as described. The nucleus and cytoplasm are identified by the nucleus detection algorithm. Blue = DAPI, green = ER

**Fig. 4:**
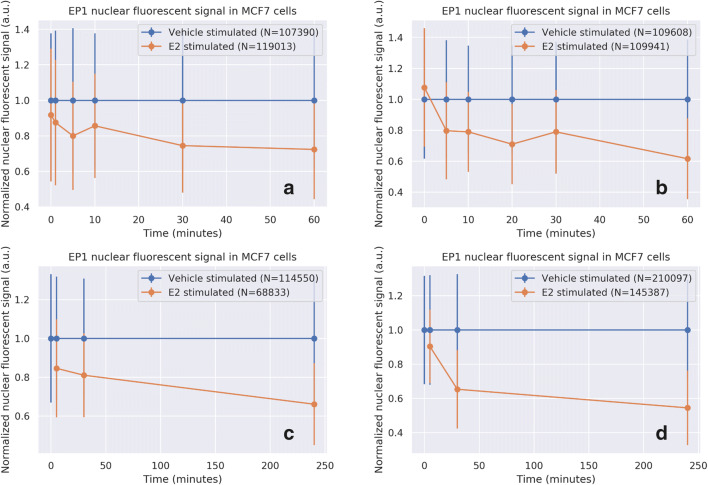

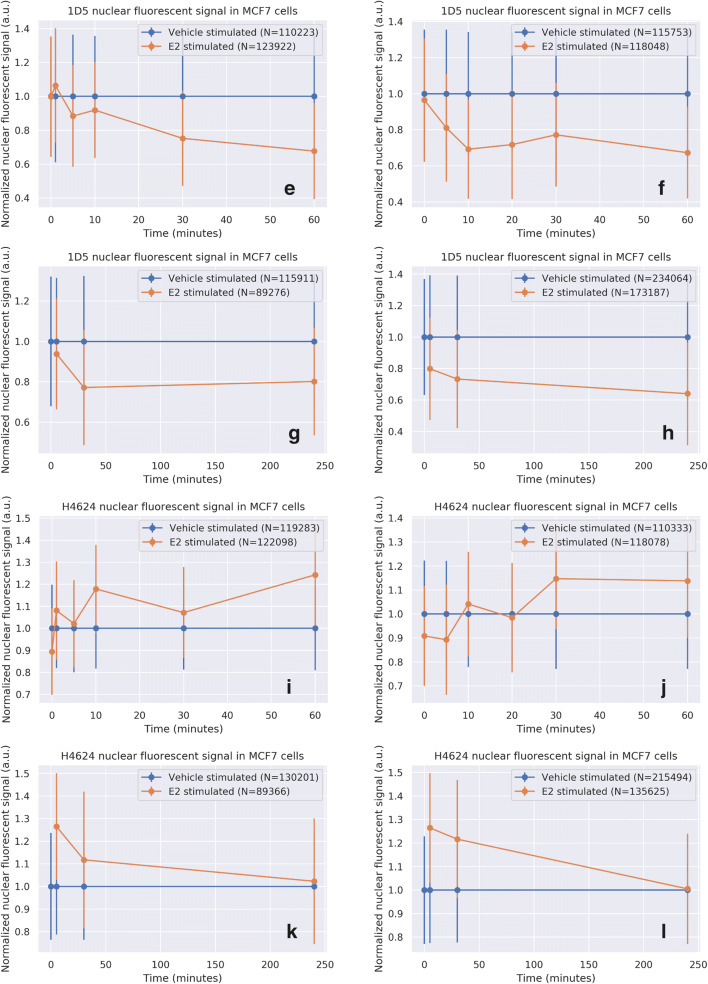
Nuclear ER staining in breast cancer cells with an inactive or active ER pathway. Estrogen-deprived MCF7 cells were treated either with vehicle (blue line) or with 10 nM E2 (orange line). At indicated time points immunofluorescent staining was performed using the indicated MoAb. Nuclear staining intensity was measured and normalized per individual cell, using the nucleus detection+ER-staining algorithm (y-axis). Exposed time is depicted on the X-axis in minutes. For each MoAb 4 independent experiments are depicted. Bars indicate the standard deviation of nuclear staining for a measurement of 10,000 to 70,000 cells **a**-**d**: EP1 MoAb; **e**-**h**: 1D5 MoAb; **i**-**l**: H4624 MoAb. N indicates the number of analysed cells

To quantify the relationship between nuclear ER staining and ER activity, immunofluorescence with EP1, 1D5, and H4624 MoAbs was performed on MCF7 and T47D cells at different time points after estradiol stimulation (5, 10, 30, and 60 min) (Fig. 4a-l). Quantification of immunofluorescent nuclear staining required image scanning and the development of software capable of recognizing the cell nuclei. The algorithm for nucleus detection functioned well on scanned slides containing stained cells, as shown in the example slide in Fig. 3.

**Fig. 5:**
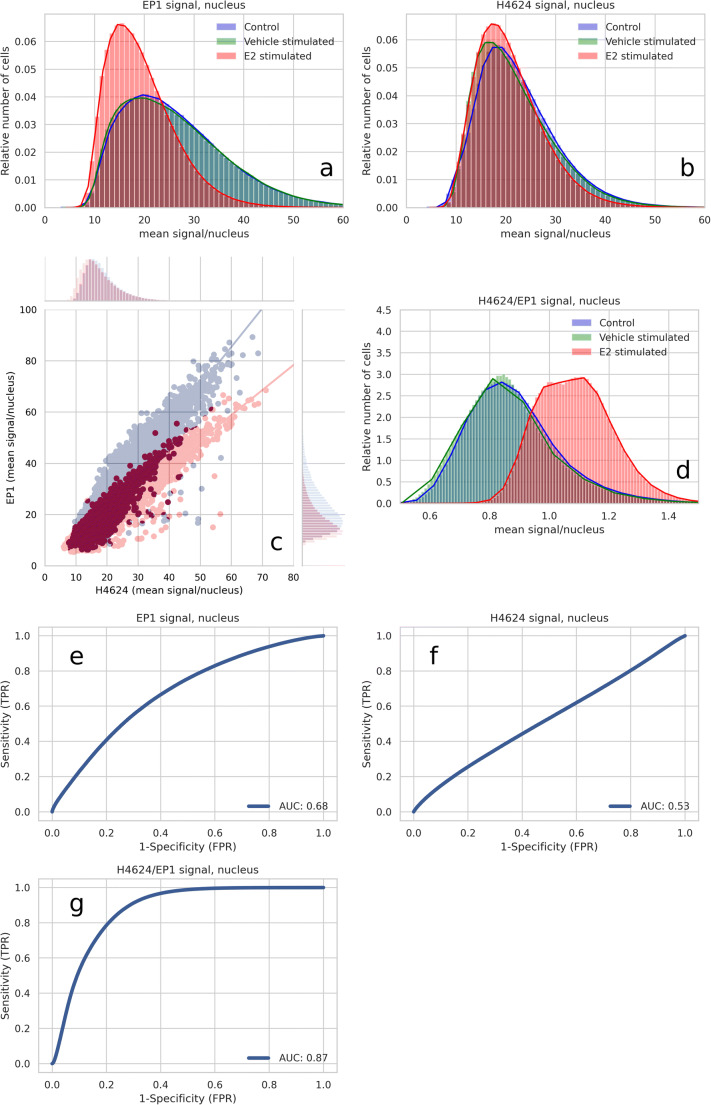
Histograms of mean nuclear fluorescent intensity signals generated using immunofluorescent staining with indicated Moabs in estrogen-deprived MCF7 cells, treated with either vehicle or estradiol for 30 min. Blue: E2-depleted MCF7 cells (*n* = 771,444 cells) (control); green: E2 depleted cells treated for 30 min with DMSO vehicle (DMSO, *n* = 758,369 cells); red: E2-depleted cells stimulated with 10 nM E2 for 30 min (*n* = 789,734 cells). Fluorescence intensity is in Arbitrary Fluorescence Units (AFUs), provided by the digital slide image scanner. *N* = 12 independent experiments, performed in duplo. **a**. EP1 MoAb; **b**. H4624 MoAb; **c**. Correlation between immunofluorescent signal intensity of H4624 and EP1 MoAb. Shifts between the two correlation plots are visualized as corresponding histograms at the top and right sides of the figure quadrant. The shift from *blue* to *red* illustrates per cell the measured decrease in nuclear staining with the EP1 MoAb following cell stimulation with estradiol, relative to nuclear staining obtained with the H4624 MoAb. **d**. Histograms of the ratio of the mean nuclear fluorescent staining intensity with EP1 and with H4624 MoAb; **e**. The sensitivity and specificity of the EP1 staining intensity when used to identify transcriptionally active nuclear ER staining, AUC (Area Under the ROC Curve) = 0.68; **f**. Sensitivity and specificity of the H4624 staining intensity when used to identify transcriptionally active nuclear ER staining, AUC = 0.53; **g**. Sensitivity and specificity of the H4624/EP1 staining intensity ratio identifying transcriptionally active nuclear ER staining, AUC = 0.87

Nucleus detection was used to quantify the nuclear fluorescence intensity per cell in a scanned image of a slide and to construct histograms from all the cells combined. Compared to nuclear ER staining intensity in estrogen-deprived cells, ER staining intensity decreased within the first five minutes of estradiol stimulation with either the EP1 or the 1D5 MoAb (Fig. 4a-d and e-h). In contrast, nuclear staining intensity with the H4624 MoAb did not decrease after cellular stimulation with estradiol (Fig. 4i-l). Similar results were obtained using the same approach with the ER-positive cell line T47D (Supplementary Fig. 1a, b and 2a, b). Use of the polyclonal ER antibody H184 resulted in staining intensity results comparable to those obtained with the H4624 MoAb and also found to be independent of the ER activity state (Supplementary Fig. 2a, b). Cell histograms illustrate in a quantitative manner that EP1 nuclear staining intensity shifted downward upon ER activation, while H4624 staining was not altered (Fig. 5a, b). Heterogeneity in nuclear ER staining was also observed between cells on the analysed slides (Fig. 5a, b).

### Prediction of ER Activity Based on Nuclear H4624/EP1 Staining Ratio

We proceeded to use our observed differences between the staining behaviours of the EP1 and H4624 MoAbs to develop a dual staining assay to predict the activity state of the ER pathway. Absolute nuclear staining intensity values are unlikely to be sufficiently predictive for ER pathway activity, due to variations in staining procedures or sample qualities for example. For this reason, the ratio H4624/EP1 was calculated between staining intensities obtained with the two MoAbs. As seen in Fig. 5d, the H4624/EP1 ratio increased in the ER active condition. Compared to individual MoAb staining (Fig. 5a, b), the H4624/EP1 ratio was better able to separate the ER-inactive condition from the ER-active condition (Fig. 5d). The expectation that the H4624/EP1 ratio would be superior in predicting ER activity was confirmed by comparing its receiver operating characteristic (ROC) curve (AUC = 0.87) with that of single MoAb EP1 (AUC = 0.68) and H4624 (AUC = 0.53) (Fig. 5e-g). The workflow steps leading to the H4624/EP1 ratio and ROC curve are illustrated in Supplementary Figs. 3 and 4. Similar results were obtained by replacing H4624 with the polyclonal antibody H184 (Supplementary Fig. 2c, d).

## Discussion

In two ER-positive breast cancer cell lines, the nuclear ER expression was measured by staining with various ER MoAbs and compared with the ER transcriptional activity measured with the mRNA-based ER pathway activity assay developed in our lab. This assay measures functional activity of the ER transcription factor as a readout of ER pathway activity [1].

We show that nuclear presence of ER in a cell is not sufficient for ER transcriptional activity, given that cells stained positive for ER in the absence of a transcriptionally active ER. This confirms earlier results obtained in clinical studies, where the ER pathway activity assay was performed on tissue samples from patients with ER-positive breast cancer. In ER-positive breast cancer, the ER pathway is shown to be either inactive or active, while an ER-inactive pathway was associated with progressive disease under neoadjuvant endocrine therapy as well as with increased incidence of recurrence under adjuvant endocrine therapy [1, 3, 11].

The estrogen receptor belongs to the nuclear receptor family of transcription factors and requires ligand-induced dimerization and recruitment of coactivator proteins to become transcriptionally active [12]. Indeed, the availability of estrogen ligand determines the activity status of nuclear ER, in line with the known mechanism of activation of the ER signalling pathway.

While all MoAbs used in this study stained nuclear ER, there were remarkable differences in staining behaviours. The level of ER staining with either EP1 or 1D5 MoAbs decreased rapidly upon transcriptional activation of ER, while staining with the H4624 MoAb appeared to be independent of ER activity. The ER activation process is associated with conformational changes in ER proteins and the binding of multiple other proteins to form an active transcription complex. Thus, we hypothesized that the single binding epitope for either EP1 or 1D5 MoAbs becomes hidden and inaccessible for antibody binding, in contrast to the binding epitope for the H4624 MoAb that clearly remains available despite changes in ER activity (Fig. 5). Nuclear ER staining by the polyclonal ER antibody H184 was also independent of the activation state of ER, providing support for the hypothesis that only a limited number of the many binding epitopes for this mixture of antibodies become hidden upon ER activation.

**Fig. 6:**
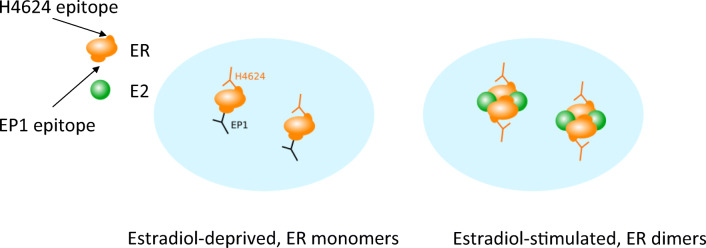
Proposed mechanism of MoAb binding in ER positive MCF7 cells with either a functionally inactive (**a**) or active (**b**) ER. A. ER is in its inactive, monomeric state when epitopes for both EP1 and H4624 MoAbs are accessible; **b**. E2 induces ER dimerization and binding of co-activators, resulting in a transcriptionally active multi-protein ER transcription factor complex. During this process, the epitope that is recognized and bound by the EP1 MoAb is obscured by ER conformational changes and ER protein-protein interactions, while the target epitope for the H4624 MoAb remains accessible

The observation that nuclear ER staining using EP1 and 1D5 MoAbs differed depending on the activity state of ER allowed for the development of a dual antibody staining assay for improved prediction of the ER activity state. The method we describe in this study requires generation of a digital image with a standard digital pathology scanner, application of a cell nucleus detection algorithm, and a *per cell* calculation of the nuclear H4624/EP1 fluorescence intensity ratio. The H4624/EP1 ratio score correlates with and predicts the probability of ER activity. In principle, the EP1 MoAb can be replaced by 1D5 or another MoAb that binds an epitope that becomes inaccessible upon ER activation. Similarly, the H4624 MoAb can be replaced by the H184 polyclonal antibody or another antibody which binds ER independent of its activity state. Translation from a fluorescent to an immunohistochemistry-based assay should also be possible. The concept that underlies the dual staining assay, that is, the use of digitized staining data obtained with two antibodies recognizing different functional states of a protein to calculate a staining intensity ratio per cell, can in principle be applied to develop staining assays for many different proteins in need of functional state characterization (Fig. 6).

Compared to the mRNA-based ER pathway activity assay, the dual staining ER activity assay has the advantage of being able to assess ER activity within preserved tissue architecture. However, it lacks the quantitative nature of the mRNA-based assay. It needs to be explored to what extent the assay is compatible with other types of sample preparation, such as formalin fixed paraffin embedded tissue [13]. The staining assay presented in this study is expected to have potential research and therapeutic value that is complementary to the mRNA-based ER pathway activity assay. For example, when the ER pathway in a tissue sample is measured as active using the ER pathway assay, the dual ER staining assay can provide an important layer of additional information on the levels of activation and localization of ER-active cells in the tissue.

## Conclusion

Routine ER staining provides insufficient information on the functional activity of the ER. A dual ER staining assay was developed which improves the ER activity assessment in cells on a scanned slide. This assay may find applications in preclinical research on the ER pathway, in development of ER activity-modifying drugs, as well as for clinical diagnostics. The concept that underlies this assay is expected to be generalizable for the development of assays with other proteins where the characterization of their functional activity state on a cell or tissue slide is of value.

## Electronic supplementary material

ESM 1 (DOCX 48 kb)

ESM 2 Supplementary Figure 1. A,B. Comparison between nuclear immunofluorescent staining intensity with EP1 and H4624 MoAbs in MCF7 and T47D breast cancer cells, treated with vehicle or E2 for 30 min. Boxplots of mean fluorescent signal intensity per cell nucleus for MCF7 cells (A) and T47D cells (B), stained with either EP1 or H4624 MoAb. C,D. Comparison of mean H4624/EP1 ratio between MCF7 (C) and T47D (D) cells. Depicted is the median, with the box indicating the 2nd and 3rd quartiles. Supplementary Fig. 2: A,B. Comparison between nuclear immunofluorescent staining with 1D5 MoAb and polyclonal ER antibody in MCF7 and T47D breast cancer cells, estradiol-deprived and either treated with vehicle or with estradiol (E2) for 30 min. Boxplots of mean fluorescent signal intensity per cell nucleus for MCF7 cells (A) and T47D cells (B), stained with either 1D5 MoAb or polyclonal antibody H184. C,D. H184/1D5 ratio in ER inactive (vehicle) and ER active (E2 stimulated) MCF7 (C) and T47D (D) cells. Depicted is the median, with the box visualizing 2nd and 3rd quartile. Supplementary Fig. 3. Estrogen deprivation/stimulation (with E2) experiments with MCF7 and T47D cells on glass coverslips. Coverslips were coated with fibronectin (FN). IF: immunofluorescence; O/N: overnight. Supplementary Fig. 4: Dual immunofluorescent staining method: An image is taken with the Digital Scanner using 3 fluorescent channels, DAPI, FITC and Cy5 (A). The DAPI channel is used to identify all the cells and define a nuclear and cytosolic compartment (B) in which H4626-FITC and EP1-Cy5 signals are quantified (C). The EP1-Cy5 staining intensity reduces upon stimulation with E2 while the H4626-FITC staining intensity is unaffected (D). Nuclear H4626-FITC signal is used as a proxy for the total number of ER molecules present in the nucleus, irrespective of ER’s activation status while the EP1-Cy5 signal is used as a proxy for the number of ER molecules that lose their EP1 binding epitope upon activation of the ER pathway. When dividing the H4626-FITC signal by the EP1-Cy5 signal, the obtained ratio is independent of the absolute amount of nuclear ER. This is hypothesized to represent the fraction of ER proteins that change conformation and available antibody-binding epitopes upon E2 binding and coactivator protein binding (and consequent ER transcriptional activation). The H4626-FITC/EP1-Cy5 ratio shows improved separation of cell populations with respectively an inactive and an active ER pathway (E). The ROC curve supports the ratio as a good indicator of ER activity (F). Supplemental Table 1. Primary and secondary antibodies used in the study. (PPTX 1697 kb)
