# Supplementary material for: A Novel Dual Antibody Staining Assay to Measure Estrogen Receptor Transcriptional Activity
Source: J Fluoresc. 2020 Nov 17;31(1):219–27. doi: 10.1007/s10895-020-02635-7 (PMC7820081; doi:10.1007/s10895-020-02635-7)
Supplement: Supplementary file 1 — (DOCX 48 kb) [file 10895_2020_2635_MOESM1_ESM.docx]

**Supplementary Methods.**

***Development of cell and nucleus recognition algorithms and nuclear ER staining quantification algorithm***

Algorithms for analysis of digital images and quantification of immunofluorescent staining intensities were developed using MATLAB 2012b (The MathWorks Inc., Natick, MA, 2000, United States). Digital image analysis consisted of: (1) image pre-processing; (2) cell nucleus detection; (3) cell membrane location estimation; and (4) quantification of immunofluorescence staining intensity in detected nuclei. In the image pre-processing step the input image is subsampled by a factor of three to reduce the computational load for the subsequent steps. The image is smoothed lightly with a Gaussian smoothing filter with a sigma of 1 to suppress sensor noise. The nucleus detection step uses the DAPI signal detection channel and consists of three steps: dynamic thresholding, cleaning, and splitting. In the first step, for reasons of varying background intensity, the DAPI channel image is thresholded dynamically, meaning that the threshold is not a single value for the entire image but is adapted for background intensity. This is accomplished by strongly smoothing the DAPI image using a Gaussian smoothing filter with a sigma of 4 to flatten out nuclear fluorescent signals while preserving the much coarser variations in background intensity. The smoothed image serves as the (dynamic) threshold for the unsmoothed DAPI image resulting in a binary image that marks nuclei with a fluorescence intensity that exceeds the dynamic threshold. The second nucleus detection step, the cleaning step, consists of removing objects from the created binary image that clearly are not nuclei or clusters of nuclei, based on morphology criteria. The final nucleus detection step splits clustered nuclei into separate ones, based on identification of a local minimum along the nuclear fusion line. To find these lines, a distance transform was applied to the inverted binary nucleus image, followed by applying a watershed algorithm to the distance-transformed image. Nuclear fusion lines were used to split up clustered nuclei into separate nuclei. The membrane estimation step uses the detected nuclei to estimate the location of the cell membranes. Once nuclei had been identified, staining intensities were quantified for each imaged cell, using separate fluorescent readout channels for dual fluorescent antibody staining. For all experiments image scanning parameters (e.g. exposure time and gain) were kept constant, enabling quantitative comparison between staining intensities across different image scans.
