# Supplementary material for: A Novel Dual Antibody Staining Assay to Measure Estrogen Receptor Transcriptional Activity
Source: J Fluoresc. 2020 Nov 17;31(1):219–27. doi: 10.1007/s10895-020-02635-7 (PMC7820081; doi:10.1007/s10895-020-02635-7)
Supplement: Supplementary file 2 — Supplementary Figure 1. A,B. Comparison between nuclear immunofluorescent staining intensity with EP1 and H4624 MoAbs in MCF7 and T47D breast cancer cells, treated with vehicle or E2 for 30 min. Boxplots of mean fluorescent signal intensity per cell nucleus for MCF7 cells (A) and T47D cells (B), stained with either EP1 or H4624 MoAb. C,D. Comparison of mean H4624/EP1 ratio between MCF7 (C) and T47D (D) cells. Depicted is the median, with the box indicating the 2nd and 3rd quartiles. Supplementary Fig. 2: A,B. Comparison between nuclear immunofluorescent staining with 1D5 MoAb and polyclonal ER antibody in MCF7 and T47D breast cancer cells, estradiol-deprived and either treated with vehicle or with estradiol (E2) for 30 min. Boxplots of mean fluorescent signal intensity per cell nucleus for MCF7 cells (A) and T47D cells (B), stained with either 1D5 MoAb or polyclonal antibody H184. C,D. H184/1D5 ratio in ER inactive (vehicle) and ER active (E2 stimulated) MCF7 (C) and T47D (D) cells. Depicted is the median, with the box visualizing 2nd and 3rd quartile. Supplementary Fig. 3. Estrogen deprivation/stimulation (with E2) experiments with MCF7 and T47D cells on glass coverslips. Coverslips were coated with fibronectin (FN). IF: immunofluorescence; O/N: overnight. Supplementary Fig. 4: Dual immunofluorescent staining method: An image is taken with the Digital Scanner using 3 fluorescent channels, DAPI, FITC and Cy5 (A). The DAPI channel is used to identify all the cells and define a nuclear and cytosolic compartment (B) in which H4626-FITC and EP1-Cy5 signals are quantified (C). The EP1-Cy5 staining intensity reduces upon stimulation with E2 while the H4626-FITC staining intensity is unaffected (D). Nuclear H4626-FITC signal is used as a proxy for the total number of ER molecules present in the nucleus, irrespective of ER’s activation status while the EP1-Cy5 signal is used as a proxy for the number of ER molecules that lose their EP1 binding epitope upon activation of the [file 10895_2020_2635_MOESM2_ESM.pptx]

## Slide 1
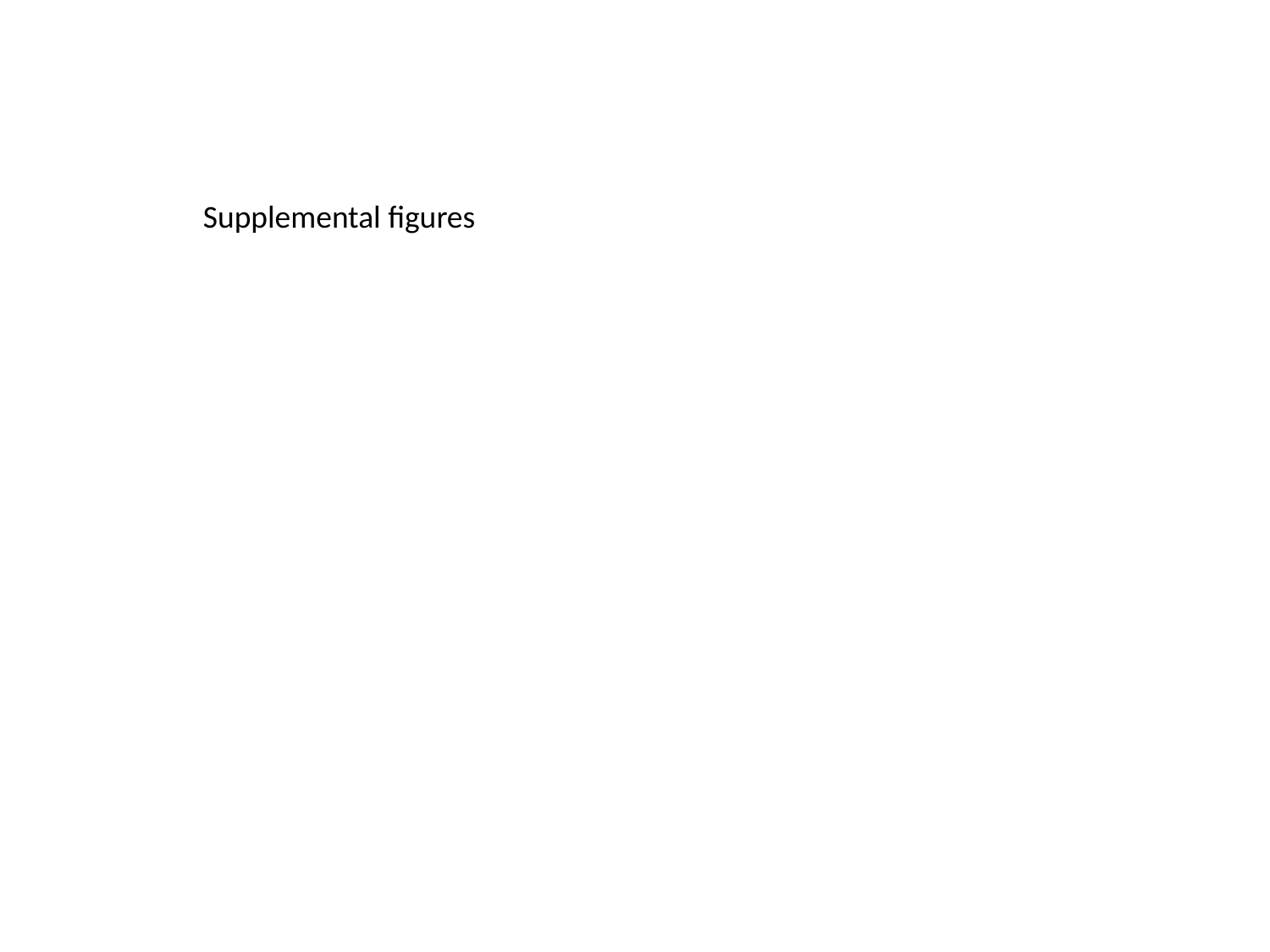

Supplemental figures

## Slide 2
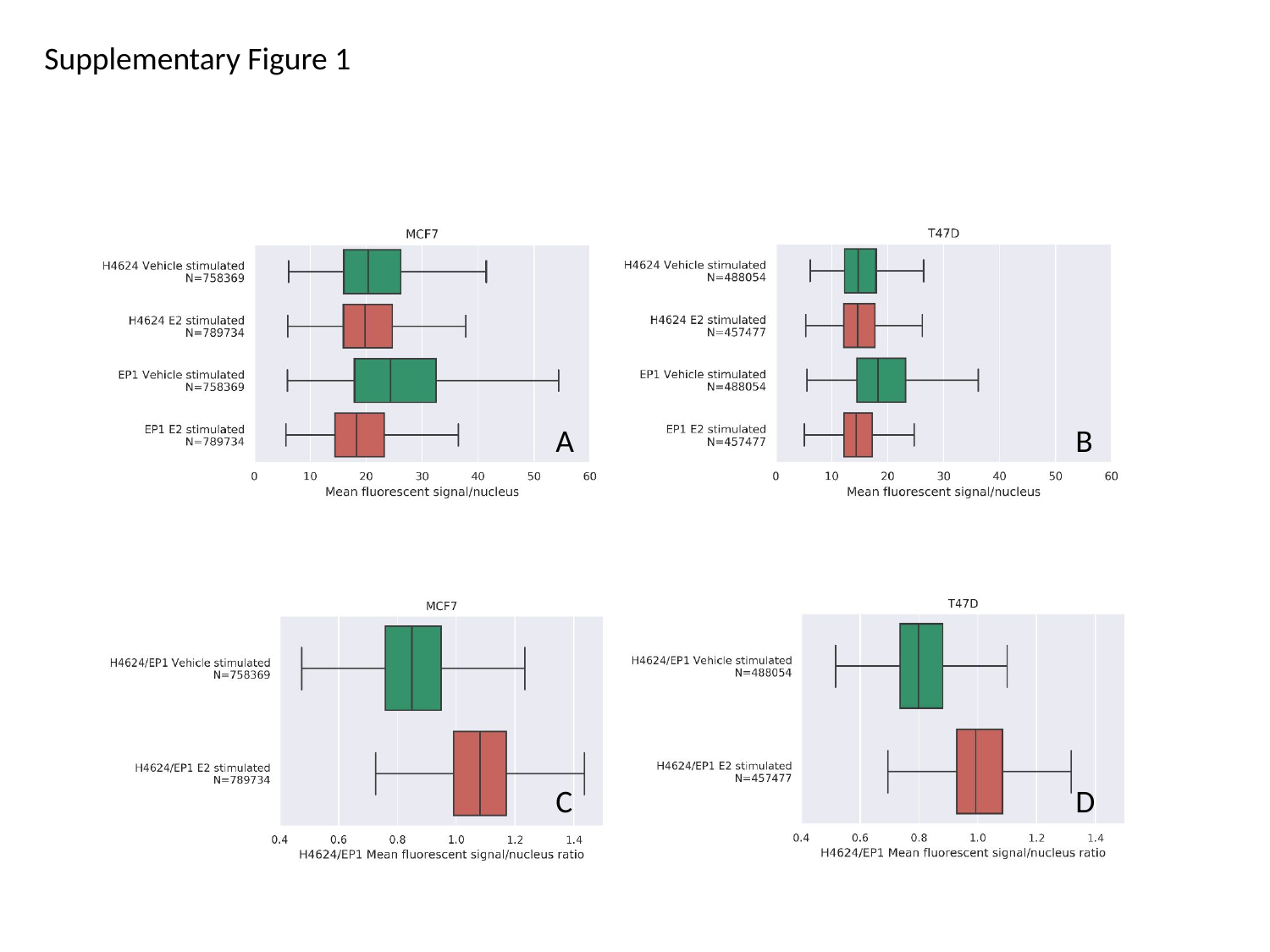

Supplementary Figure 1
A
B
C
D

## Slide 3
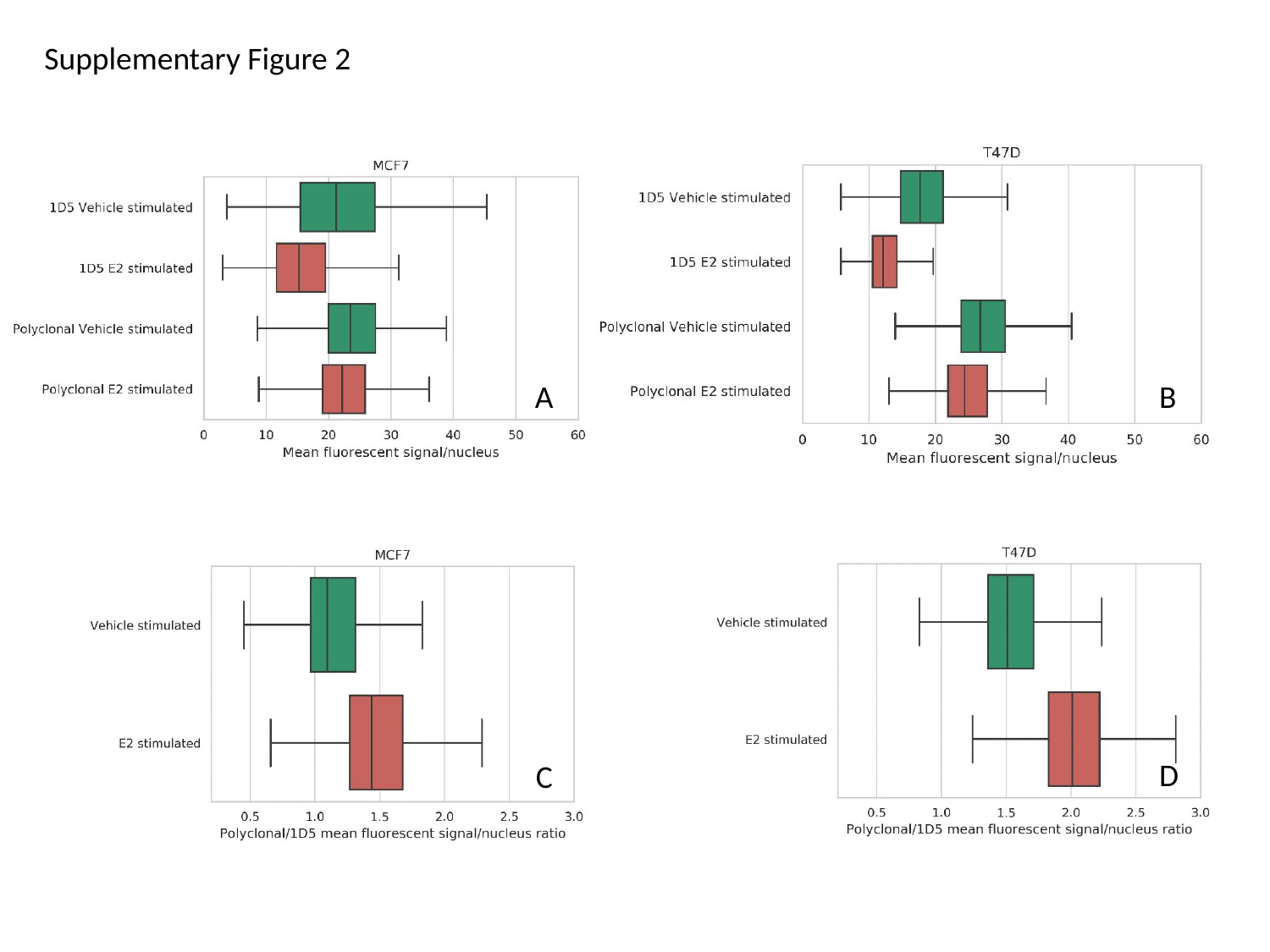

Supplementary Figure 2
A
B
D
C

## Slide 4
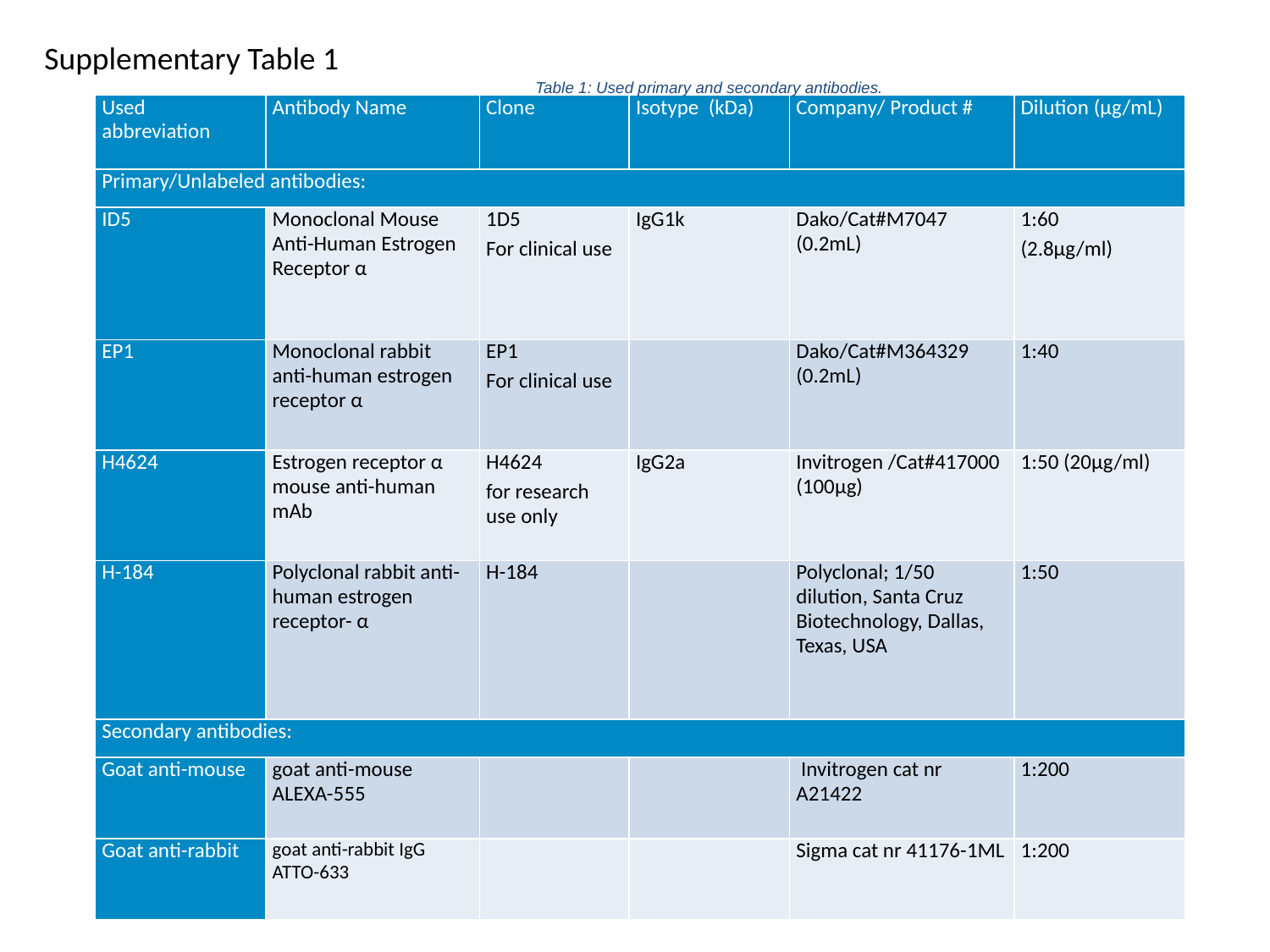

Supplementary Table 1
Table 1: Used primary and secondary antibodies.
| Used abbreviation | Antibody Name | Clone | Isotype (kDa) | Company/ Product # | Dilution (µg/mL) |
| --- | --- | --- | --- | --- | --- |
| Primary/Unlabeled antibodies: | | | | | |
| ID5 | Monoclonal Mouse Anti-Human Estrogen Receptor α | 1D5 For clinical use | IgG1k | Dako/Cat#M7047 (0.2mL) | 1:60 (2.8µg/ml) |
| EP1 | Monoclonal rabbit anti-human estrogen receptor α | EP1 For clinical use | | Dako/Cat#M364329 (0.2mL) | 1:40 |
| H4624 | Estrogen receptor α mouse anti-human mAb | H4624 for research use only | IgG2a | Invitrogen /Cat#417000 (100µg) | 1:50 (20µg/ml) |
| H-184 | Polyclonal rabbit anti-human estrogen receptor- α | H-184 | | Polyclonal; 1/50 dilution, Santa Cruz Biotechnology, Dallas, Texas, USA | 1:50 |
| Secondary antibodies: | | | | | |
| Goat anti-mouse | goat anti-mouse ALEXA-555 | | | Invitrogen cat nr A21422 | 1:200 |
| Goat anti-rabbit | goat anti-rabbit IgG ATTO-633 | | | Sigma cat nr 41176-1ML | 1:200 |

## Slide 5
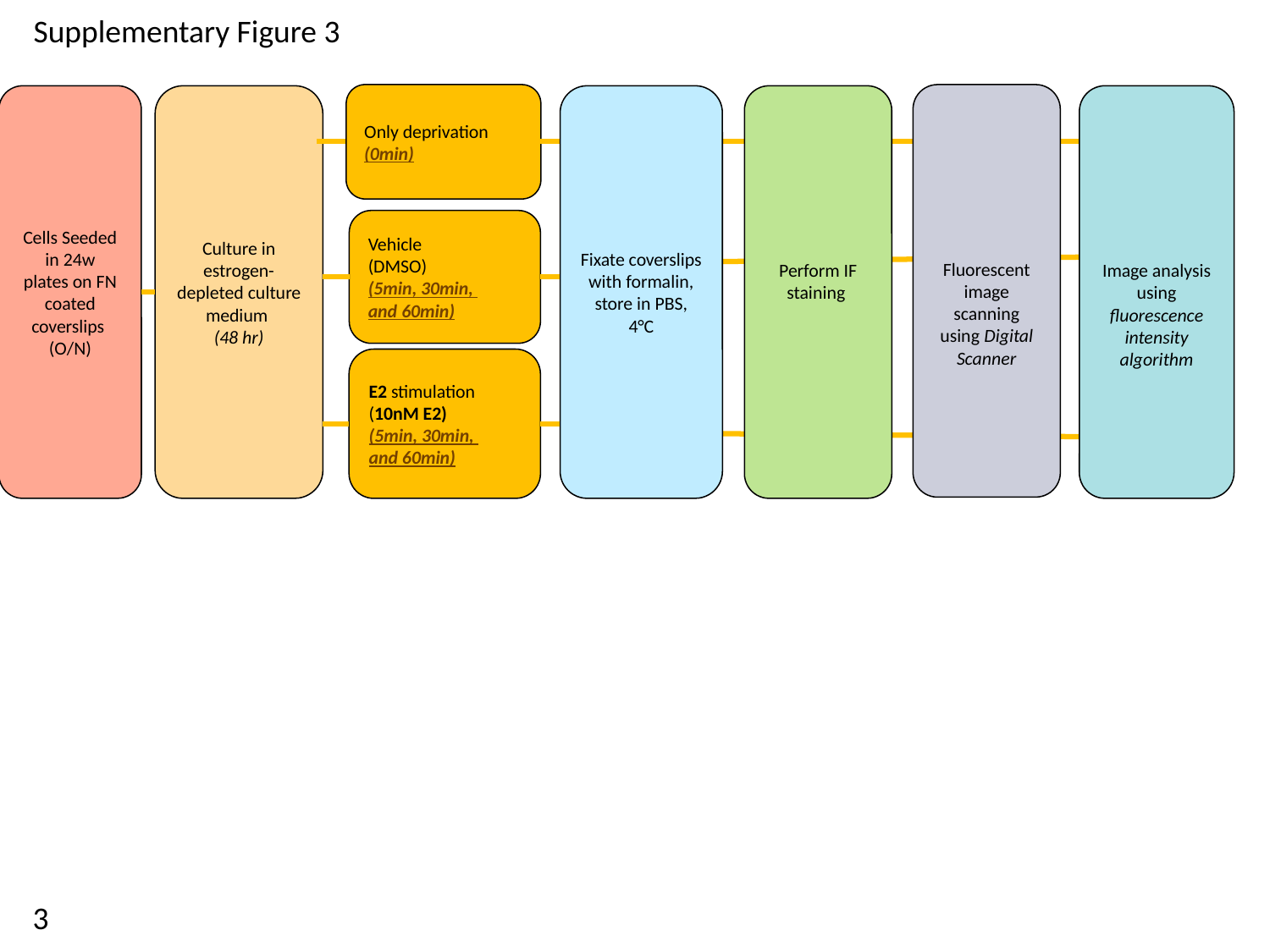

Supplementary Figure 3
Only deprivation
(0min)
Fluorescent image scanning using Digital Scanner
Cells Seeded in 24w
plates on FN coated coverslips
(O/N)
Culture in estrogen-depleted culture medium
(48 hr)
Fixate coverslips with formalin, store in PBS, 4°C
Perform IF staining
Vehicle (DMSO)
(5min, 30min,
and 60min)
E2 stimulation (10nM E2)
(5min, 30min,
and 60min)
Image analysis using fluorescence intensity algorithm
3

## Slide 6
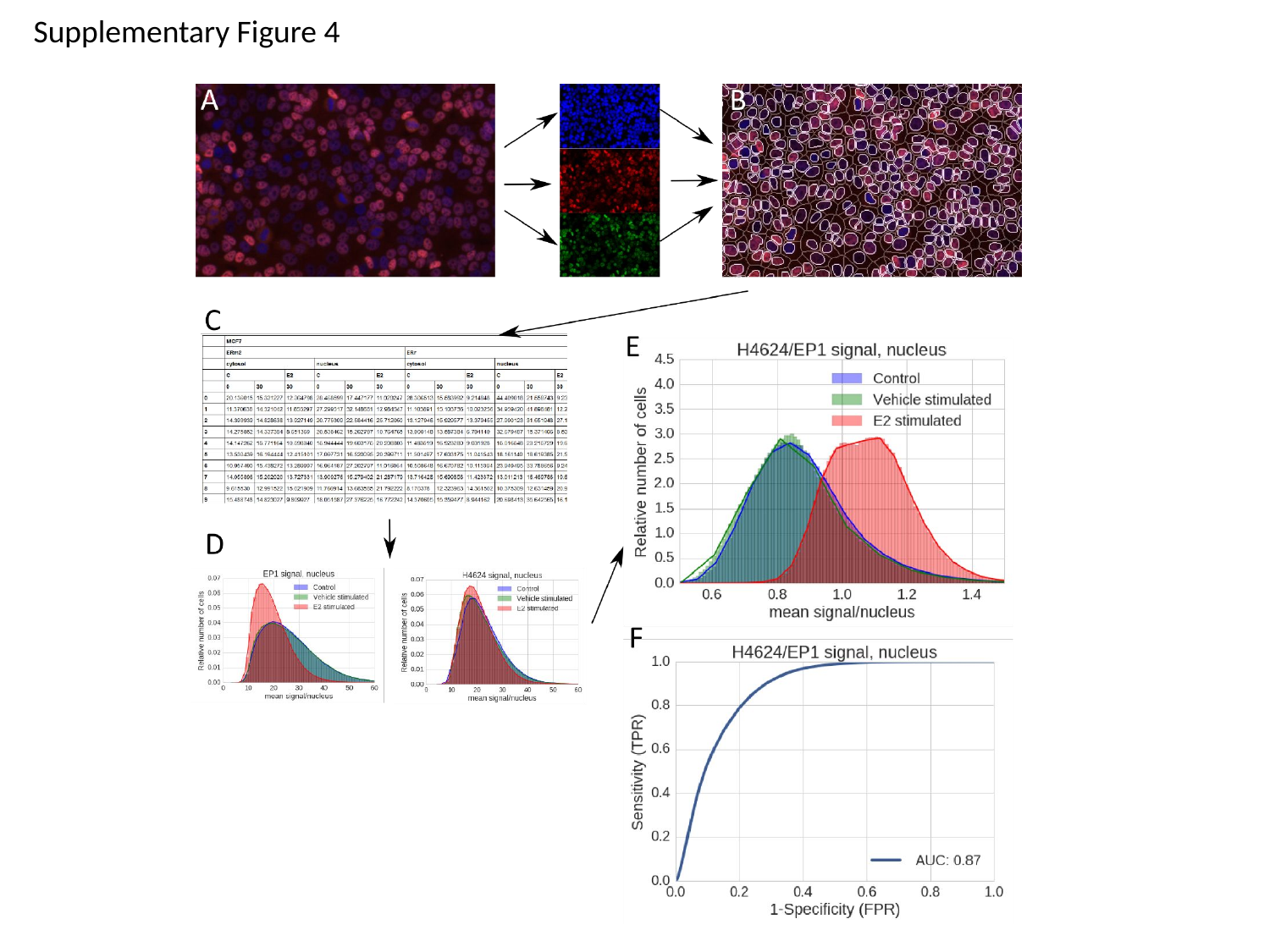

Supplementary Figure 4
